# Using PVA and captive breeding to balance trade-offs in the rescue of the island dibbler onto a new island ark

**DOI:** 10.1038/s41598-022-14150-9

**Published:** 2022-07-13

**Authors:** Zahra Aisya, Daniel J. White, Rujiporn Thavornkanlapachai, J. Anthony Friend, Kate Rick, Nicola J. Mitchell

**Affiliations:** 1 School of Biological Sciences, The University of Western Australia, 35 Stirling Highway, Crawley, WA 6009 Australia; 2 Department of Biodiversity, Conservation and Attractions, Locked Bag 104, Bentley Delivery Centre, Perth, WA 6983 Australia; 3 Department of Biodiversity, Conservation and Attractions, 120 Albany Highway, Albany, WA 6330 Australia

**Keywords:** Genetic markers, Conservation biology, Ecological genetics, Population dynamics

## Abstract

In the face of the current global extinction crisis, it is critical we give conservation management strategies the best chance of success. Australia is not exempt from global trends with currently the world’s greatest mammal extinction rate (~ 1 per 8 years). Many more are threatened including the dibbler (*Parantechinus apicalis*) whose remnant range has been restricted to Western Australia at just one mainland site and two small offshore islands—Whitlock Island (5 ha) and Boullanger Island (35 ha). Here, we used 14 microsatellite markers to quantify genetic variation in the remaining island populations from 2013 to 2018 and incorporated these data into population viability analysis (PVA) models, used to assess factors important to dibbler survival and to provide guidance for translocations. Remnant population genetic diversity was low (< 0.3), and populations were highly divergent from each other (pairwise F_ST_s 0.29–0.52). Comparison of empirical data to an earlier study is consistent with recent declines in genetic diversity and models projected increasing extinction risk and declining genetic variation in the next century. Optimal translocation scenarios recommend 80 founders for new dibbler populations—provided by captive breeding—and determined the proportion of founders from parental populations to maximise genetic diversity and minimise harvesting impact. The goal of our approach is long-term survival of genetically diverse, self-sustaining populations and our methods are transferable. We consider mixing island with mainland dibblers to reinforce genetic variation.

## Introduction

In Australia, translocations are widely used to improve the status of threatened mammal species by increasing the number of self-sustaining populations^1,2^, and safeguarding species on predator-free onshore and offshore havens^3–5^. Maintaining genetic diversity is a key component to successful translocation programs^2,6^, and IUCN/SSC guidelines^7^ recommend that individuals selected for translocations should provide adequate genetic diversity, and come from geographically close origins and comparable habitats to the intended destination. Further, it has been suggested that founders should capture 90–95% of genetic diversity of source populations^7–9^, and should be genetically compatible if they are mixed from multiple sources^10^.

Gaining access to ideal founder populations may not be possible if a species is restricted to offshore islands^11,12^. Island populations are often isolated with no immigration and constrained effective population sizes lead to low genetic diversity. Using island sources as founder populations in translocations may compound effects such as population bottlenecks in the translocated population. Depending on the number of individuals harvested (which determines the narrowness of the bottleneck), there can be increased inbreeding, loss of evolutionary potential and rapid genetic divergences from the source population(s)^6,13,14^. There can also be a negative impact on the source population(s)^15^, which is particularly relevant for species whose remnant ranges are restricted to just a few locations. Harvesting too many individuals from a population can alter population subdivision, reduce genetic variation, induce selective genetic changes and lead to irreversible population decline^16^.

Captive breeding has played critical roles in conservation management of threatened species when remnant source populations are of limited or uncertain size, and mitigates the inherent risk of extinction due to over-harvesting for wild-to-wild translocations (e.g. the Californian condor^17^ and the western swamp tortoise^18^). While captive breeding programs provide relatively benign environments that allow populations to thrive and persist over time, issues associated with captive breeding include high cost, the possibility of individuals adapting to captivity and being unsuited to release, and the potential for disease outbreaks^19,20^. Nevertheless, captive breeding programs play an important role in conservation management of many species, but their implementation should be carefully designed^19^.

One way to counterbalance the loss of genetic diversity when establishing new populations is to use multiple source populations. Genetic mixing of source populations can increase heterozygosity, increase adaptive potential and mask deleterious inbreeding effects in translocated populations^21,22^. However, there are risks associated with such mixing, which include pre-zygotic isolation (e.g. morphology, behaviour, and gametic incompatibilities) and post-zygotic isolation (e.g. abnormal chromosomal structure and harmful epistatic interaction between parental alleles) which can result in a lack of interbreeding or fitness decline of admixed progeny^10,23,24^. For example, crossing populations that are adapted to local habitats can disrupt important gametic associations and dilute adapted alleles, resulting in hybrids with lowered fitness, referred to as outbreeding depression^25–28^. All these factors can reduce the effective population size of a newly established population, and subsequently lead to a detrimental loss of genetic variation^14^.

**Figure 1:**
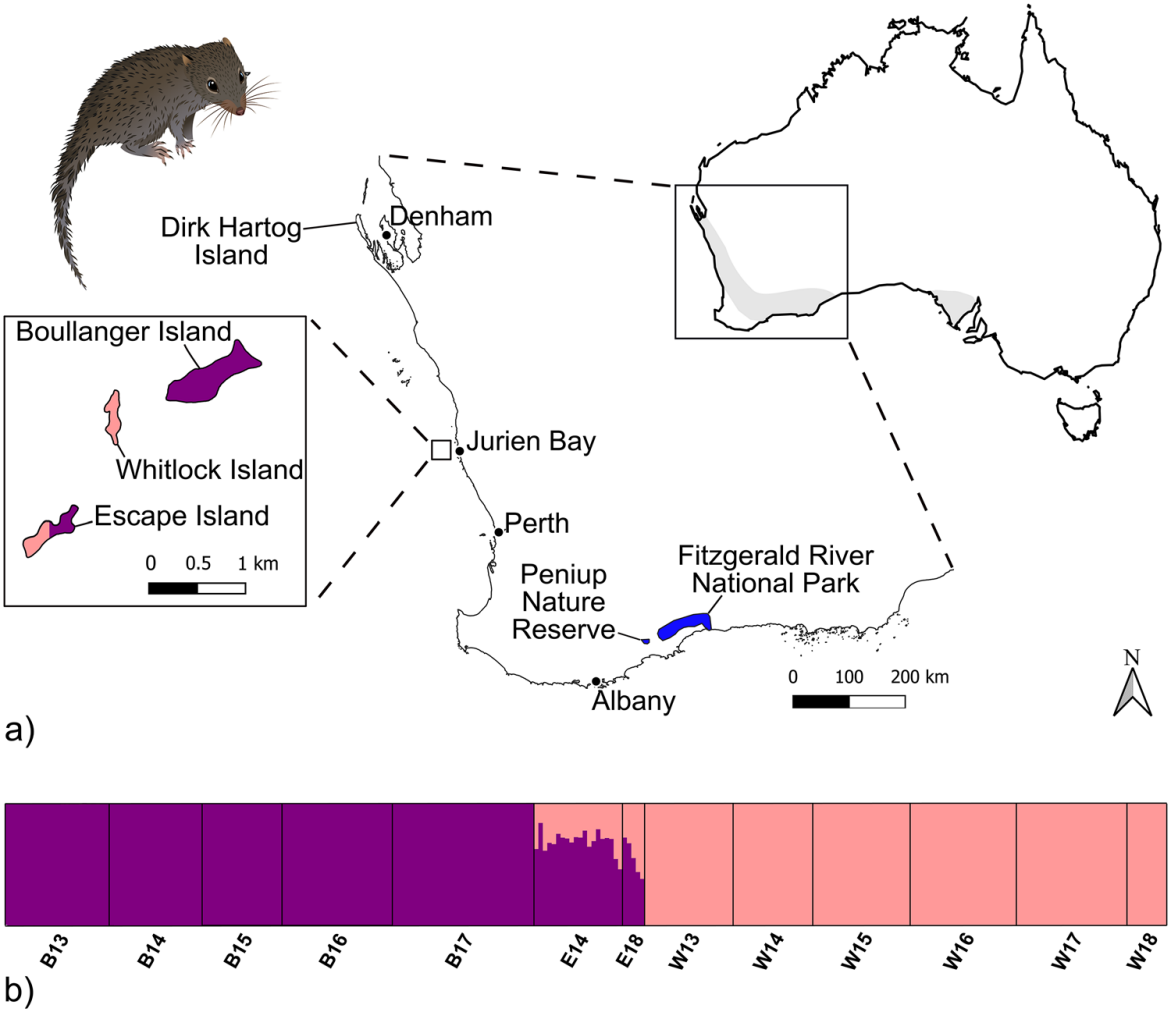
(**a**) Current distribution of island dibblers in Jurien Bay and historic distribution of dibblers in Australia (inset). Dirk Hartog Island, the location of the new translocation, is also shown. (**b**) STRUCTURE analysis showing the number of genetic clusters (*K* = 2) within island dibblers and the level of admixture in the Escape Island population. Black lines separate islands and collection years. *B* Boullanger, *W* Whitlock, *E* Escape. Dibbler image sourced from Creazilla under an Attribution 4.0 International (CC BY 4.0) license. Image can be found at https://creazilla.com/nodes/64031-dibbler-clipart.

The dibbler (*Parantechinus apicalis*) is a small (40–125 g) dasyurid marsupial once widely distributed in Australia (Fig. 1) but has declined dramatically due to introduced predators such as foxes (*Vulpes vulpes*) and feral cats (*Felis catus*), inappropriate fire regimes, habitat degradation due to dieback (*Phytophthora cinnamomi*) disease, and competition with house mice (*Mus musculus*)^29^. Dibblers are now restricted to the southwest of Australia^30–33^ occurring naturally on mainland Australia in the Fitzgerald River National Park (~ 3000 km^2^), and on two small islands, Boullanger and Whitlock Islands off the mid-west coast of Western Australia (Fig. 1). The species is listed as Endangered under Australia’s environmental legislation, the Environment Protection and Biodiversity Conservation (EPBC) Act 1999, and on the International Union for Conservation of Nature (IUCN) Red List of Threatened Species^34^.

To prevent further population declines, five translocations have been implemented since 1998 using captive bred dibblers sourced from island and mainland populations^4,35^. Only three have been successful, including a translocation to Escape Island in 1998 using 88 captive-bred dibblers sourced from Boullanger and Whitlock Islands^36^. In 2001, another successful translocation to Peniup Nature Reserve was achieved using 235 captive-bred dibblers sourced from mainland populations^37^, and in 2015, 80 captive-bred mainland dibblers were introduced to Gunton Island off the south coast of Western Australia^35^. Although in its early stages, recent monitoring suggests the Gunton Island population has successfully established (J.A. Friend, personal observation). However, despite these efforts, fewer than 1000 individuals, including those in translocated populations, remain in the wild^34^.

The reintroduction of dibblers to Dirk Hartog Island, a large island (62,000 ha) off the coast of Western Australia (Fig. 1), is a key component of a major ecological restoration project ‘*Return to 1616’* led by the Western Australian Department of Biodiversity, Conservation and Attractions (DBCA)^38^. Following eradication of feral cats and goats, 11 mammal and one bird species are being translocated to Dirk Hartog Island between 2018 and 2030, with the aim of restoring former faunal biodiversity and ecosystem processes. Dibblers are being sourced from the three Jurien Bay islands but are first being captivity-bred at Perth Zoo to enhance founder numbers. However, as the Jurien Bay island populations are small (10–30 adults) and subject to large fluctuations, they are vulnerable to extirpation (J.A. Friend, unpublished data and^39^), especially if harvesting pressure is too high. Further, it is currently unknown how many founders are required to establish a population on Dirk Hartog Island.

Population viability analyses (PVA) are widely used in planning and evaluating conservation actions for threatened populations^40–43^, and can assist with managing finely balanced trade-offs when planning translocations^44^. In this study we use PVA to explore management options, and their subsequent impact on genetic diversity and survival, for the island dibbler. We first quantify changes in genetic variation in the Jurien Bay island populations from 2013 to 2018. We then incorporate these genetic data into population viability analysis (PVA) models which were developed to explore various harvesting options for a captive breeding program, provide guidance for translocations to Dirk Hartog Island and assess universal factors important to dibbler survival. Our overarching objective is to maximise genetic diversity and long-term survival in new populations while minimising impact on the source populations.

## Materials and methods

### Study species

Dibblers have a predominantly insectivorous diet, a crepuscular nature and inhabit areas of dense unburnt vegetation^29,30,36^. Female dibblers have an annual oestrous period, breed in autumn and carry a single brood of up to eight pouch young^33,45–47^. On Boullanger and Whitlock Islands, males die after the first mating season in some years^31^. Facultative male die-off occurs more often on Boullanger Island than Whitlock Island^31^, and has not been observed on Escape Island^36^. In mainland populations, males survive well into their second year^29^, and no male die-off has been observed in captivity^31,33,45,47^. While facultative male die-off appears to be a consequence of an extreme mating strategy in response to highly seasonal and limited breeding periods^48^, reduced availability of food and nutrients may increase its frequency on Boullanger Island^49^.

### Study sites and tissue collection

This study focuses on three Jurien Bay island populations: two parental—Boullanger Island (35 ha) and Whitlock Island (5.4 ha), and one translocated—Escape Island (10.5 ha) (Fig. 1a). Tissue samples (ear notches) from each island (Boullanger Island, N = 119; Whitlock Island, N = 118; Escape Island, N = 25) were collected non-fatally as part of routine monitoring between 2013 and 2018 and stored at room temperature in solutions of 20% dimethyl sulfoxide (DMSO) or 90% ethanol.

### DNA extractions and genotyping

DNA was extracted using a salting out method^50^ in 340 µL TNES buffer, 10 µL proteinase-K, and 3 µL RNase. DNA concentrations were measured from 20 samples chosen at random using a Qubit Fluorometer, and DNA quality was checked by running gel electrophoresis. Polymerase chain reaction (PCR) was completed using a QIAGEN Multiplex PCR PlusKit, with a one in ten dilution of all DNA samples and 14 microsatellite primer pairs (reaction details are provided in Supplementary Table S1). PCR amplification was done using Eppendorf Mastercycler X50 and Eppendorf Mastercycler nexus thermocyclers, with the following cycling conditions: 95 °C for 15 min; a total of 30 cycles of 94 °C for 30 s, various annealing temperatures for different primers for 90 s (Supplementary Table S1), 72 °C for 60 s; and concluded with 60 °C for 30 min followed by 25 °C for 60 s. PCR products were prepared in HiDi Formamide and GeneScan 500 LIZ size standard before being analysed with an ABI 3730 sequencer by the Western Australian State Agricultural Biotechnology Centre (SABC).

Scoring of genotypes was completed using the software GeneMapper 5 (Applied Biosystems). For validation and standardisation of genotypes, samples from Boullanger Island (n = 5), Whitlock Island (n = 5), and Escape Island (n = 6) populations from 2012 or earlier were compared to an earlier cohort^39^. Current genotypes were compared to original genotypes for these samples and allele calls were standardised across all loci for temporal comparisons.

### Genetic analyses

To assess if null alleles were present within the populations, all loci were analysed with MICROCHECKER v2.2.3^51^. The genetic diversity of each population was assessed, including mean number of alleles per locus (NA), allelic richness (an estimate of allele number per locus corrected for sample size), observed (H_O_) and expected (H_E_) heterozygosity. Deviations from Hardy–Weinberg Equilibrium were assessed by calculating inbreeding coefficients (F_IS_) for each population. Positive F_IS_ values represent a deficit in heterozygosity while negative F_IS_ values represent an excess of heterozygosity. These metrics were analysed using FSTAT v2.9.3.2^52^ and GENALEX v.6.503^53,54^. Differences in allelic richness and heterozygosity values between populations were tested with Wilcoxon’s rank sum test in R v3.5.1 statistical package^55^, with samples paired by locus.

To measure genetic distances between populations, pairwise F_ST_ values were calculated using FSTAT v2.9.3.2^52^ and pairwise Jost’s D values were calculated in GENALEX v.6.503^53,54^. Pairwise F_ST_ measures genetic fixation or the amount of genetic drift between populations, whereas pairwise Jost’s D measures allelic differentiation. For both, values can range from zero (low divergence) to one (high divergence). Clustering analysis using the program STRUCTURE v2.3.4^56^ was completed to visualise the genetic composition of the translocated population on Escape Island. As previous work has shown that Boullanger and Whitlock Islands are genetically divergent^39^, suggesting no gene flow between islands since rising sea levels separated them, we assumed allele frequencies were uncorrelated in our models. In contrast, Escape Island is an admixed population, and so we compared models that both included and excluded admixture. The number of clusters (K) was set from one to ten, and ten replicates were run per K tested, over 100,000 steps of the Monte Carlo Markov chain after a burn-in length of 10,000 steps. To confirm the best value for K, ΔK was estimated in STRUCTURE HARVESTER^57^, where the largest ΔK value indicated the K value which was the best fit^58^.

Estimates of effective population size (Ne), defined as the size of an ideal population that will show an equal rate of genetic drift as the observed population, were generated in NeEstimator v2.1^59^. A linkage disequilibrium model with random mating was selected, and the lowest allele frequency was set to 0.05^60^. To detect the probability of a recent bottleneck event occurring within populations, data were analysed in BOTTLENECK v.1.2.02^61^, using the two-phase model (TPM) with the probability of the stepwise mutation model (SMM) set to 95% and variance set to 12, as recommended by Piry et al.^62^. A one-tailed Wilcoxon sign rank test was used to determine excess heterozygotes.

Individual heterozygosity, or the number of heterozygous loci within each individual, is a potential indicator of an individual’s fitness and was measured in GENALEX v.6.503^53,54^, and reported as the mean per population. To determine if pairs of individuals had alleles identical by descent (IBD), Queller and Goodnight’s^63^ pairwise relatedness (*r*) was estimated in GENALEX v.6.503^53,54^. Within-group means and 95% confidence intervals for each population were calculated using 999 permutations and 1000 bootstraps, respectively. Differences within populations across years were tested with Wilcoxon’s rank sum test in R v3.5.1^55^.

### Population viability analysis and sensitivity analysis

Population viability analysis was conducted in VORTEX v.10.3.2^64^. VORTEX is an individual-based PVA program that utilises species life history traits and stochastic environmental factors to predict outputs such as the probability of extinction, population growth rate and genetic diversity^65^. A baseline PVA model for island dibblers^39^ was refined by consulting experts (J.A. Friend, personal observation) and literature^31,32,36,46^. A summary of demographic parameters used in models is provided in Table 1 and further justification in Supplementary Table S2. Drought was incorporated as a catastrophic event as it drives population dynamics of other dasyurid species, such as the agile antechinus and the brush-tailed phascogale^66,67^. Meteorological data indicate that the Jurien Bay area has periods of low rainfall at an average frequency of approximately eight years^68^. Thus, catastrophes at eight-year intervals that reduced reproduction and survival by 70% were implemented (Table 1). Population projections were for 100 years, and all models ran 500 replicates. We also ran several scenarios with 1000 replicates to compare performance, with negligible differences in results other than slightly smaller standard errors. Hence, considering the number of scenarios that were run in this study, we chose to run 500 replicates for all scenarios.

**Table 1:** Demographic parameters for the dibbler population on Boullanger Island used for population viability analysis.

Parameter	Value
Reproductive system	Polygynous^a^
Inbreeding depression	NA
Age of first offspring	1 (10 months)^b^
Maximum age of reproduction	3^b^
Maximum lifespan	3^b^
Maximum no. of broods per year	1^a^
Maximum no. of progeny per brood	8^a^
Sex ratio at birth (% in males)	49.7^c^
% Adult females reproducing	90^d^
Mean (± SD) no. of progeny per brood	7.4 ± 0.1^e^
Mortality (± SD)	
0–1 years of age	59% ± 10^f^
> 1 years of age	♂: 35% (with 8-year facultative semelparity) ± 10^g^
	♀: 35% ± 10^c^
Catastrophe	1
Frequency of catastrophe (%)	12.5^g^
Severity (proportion of normal values)	
Reproduction	0.3^g,h^
Survival	0.3^g,h^
Initial population size	84^c,f^
Population carrying capacity (± SD)	100 ± 13^c^
Years modelled	100
No. of iterations	500

NA denotes not applicable. Superscripts denote sources of data. Justifications of demographic parameters for all modelled populations are presented in Online Resource 1. ^a^Lambert and Mills^46^; ^b^Mills and Bencini^31^; ^c^Friend, pers. obs.; ^d^Moro^36^; ^e^Mills et al.^32^; ^f^Calculated—see Online Resource 1; ^g^Parrott et al.^66^; ^h^Rhind and Bradley^67^.

Sensitivity analyses, defined as the evaluation of how changes to life-history traits affect population growth or long-term viability^41,69^, were implemented on the baseline model. Each parameter was tested sequentially while keeping all other parameters constant and included mortality rates for juveniles and adults (0% to 100%, in increments of 5%), population carrying capacity (0 to 300, in increments of 20), founder number (10 to 100 in increments of 10) and frequency of droughts (0% to 20% probability in increments of 2.5%).

#### Validation of baseline model

After sensitivity testing, a best-performing (i.e. most demographically realistic) baseline model was used to estimate current population sizes, genetic diversity, and allele frequencies based on census data available for 2012^39^. Initial population sizes were set to 68, 29, and 26 for Boullanger, Whitlock, and Escape Islands respectively. Simulations were run for six years (500 replicates), and predictions from the model for 2018 were compared to empirical data collected in 2017/2018. This allowed for evaluation of how well the optimised baseline model captured short term (and by extrapolation, long term) viability and genetic diversity within the island populations.

#### Optimal harvesting models and translocation scenarios

One captive breeding population and one new population on Dirk Hartog Island were simulated, to reflect current management actions. As the Perth Zoo can accommodate ten dibbler breeding pairs (Cathy Lambert, pers. comm.) 11 scenarios were simulated to determine the ideal harvest design to provide ten males and ten females for captive breeding, without detrimentally affecting the populations on Boullanger and Whitlock Islands. These scenarios ranged from harvesting ten males and ten females from one island only, to harvesting five males and females from each island. A further 11 scenarios were simulated as above, using Escape and Whitlock Islands as source populations.

To model a translocated population on Dirk Hartog Island, the same parameters were used for the translocated population established on Escape Island, but with a much larger estimated carrying capacity of 10,000 based on land area. As female dibblers can produce a maximum of eight offspring per brood, and Perth Zoo plans to conduct captive breeding for two years, up to 160 dibblers could be available for translocation to Dirk Hartog Island and will be released over two consecutive years (Saul Cowen, pers. comm.). Allowing for some captive mortality and retention of adults for a second breeding season, release groups of 30, 40, 50, 60 and 70 juveniles per year were modelled to determine the impact of varying founder size on survival probability and genetic diversity.

#### Modelling the current captive-breeding and release program

In 2018, nine dibblers from Whitlock Island (five females, four males) and five dibblers from Escape Island (two females, three males) were captured for Perth Zoo’s latest captive breeding program. To maximise outcomes of the current captive breeding program, we modelled release groups of 20 (to account for the possibility of fewer dibblers being born in captivity than expected), 30, 40, and 50 juveniles per year at Dirk Hartog Island. While seven female dibblers can produce up to 112 dibblers over two years, we chose lower numbers to account for some mortality and animal retention for breeding. We made the same assumptions as in previous models and compared outcomes to the optimal translocation scenarios.

### Ethics approval

Permission to collect samples was granted by the Department of Biodiversity Conservation and Attractions Animal Ethics Committee (Approval Numbers 2012-62, 2015-54 and 2018-44G), and all methods were performed in accordance with relevant guidelines and regulations.

### Consent for publication

All authors give consent for this publication.

## Results

### Genetic analysis of island dibblers

The percentage of polymorphic loci in each population ranged from 7.1% (1/14) in Whitlock Island, to 64.3% in Boullanger Island (9/14) and 85.7% (12/14) in Escape Island. Since 2013, two loci had become monomorphic in two populations (locus Pa2D4 in the Boullanger Island population, and locus 4.4.10 in the Whitlock Island population). Analysis of markers in MICROCHECKER confirmed that no loci contained null alleles.

**Figure 2:**
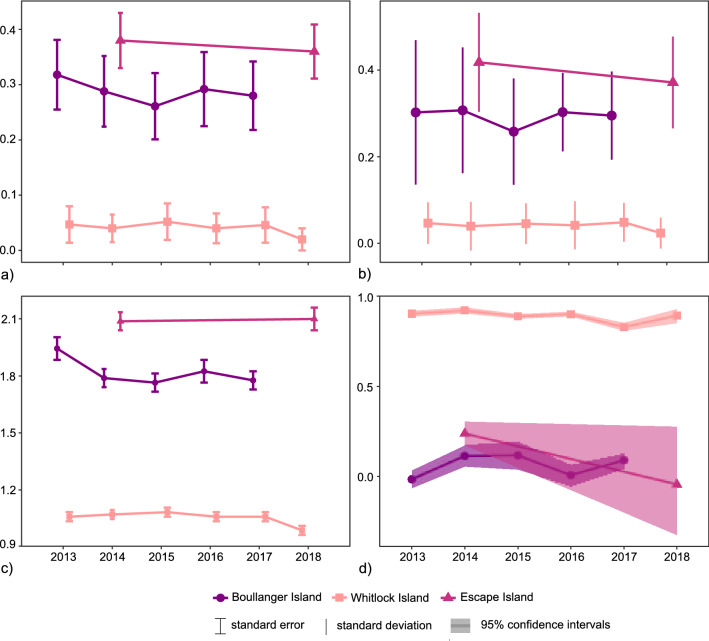
Changes in genetic diversity in island dibbler populations over time. (**a**) Expected heterozygosity, (**b**) individual heterozygosity, (**c**) allelic richness, (**d**) relatedness.

Overall, the Escape Island population had the highest allelic and genetic diversity (Table 2 and Fig. 2). While there was a trend for greater observed heterozygosity compared to expected heterozygosity in Boullanger Island and Escape Island cohorts, differences were non-significant. All Whitlock Island cohorts had identical observed and expected heterozygosities. Pairwise comparisons indicated that allelic richness, H_O_ and H_E_ values in the Whitlock Island population were significantly lower than both Boullanger and Escape Island populations (Wilcoxon’s rank sum test; *P* < 0.05). In contrast, there were no significant differences in allelic richness, H_O_, or H_E_ values between Boullanger and Escape Island populations. Inbreeding coefficients (F_IS_) ranged between – 0.14 to 0.11, but no F_IS_ value was significantly different from zero after correction for multiple comparisons. Mean values of individual heterozygosity were greatest in Escape Island, lowest in Whitlock Island and although these appeared to decline over time in all three populations, differences between the latest and earliest cohorts were not significant (Fig. 2b and Supplementary Fig. S1). Overall, population mean values of Queller and Goodnight’s pairwise relatedness (r) across multiple years in the Whitlock Island population were similar to a full-sib relationship (r = 0.83 to 0.92), which was substantially higher than Boullanger Island (r = – 0.02 to 0.12) and Escape Island (r = – 0.05 to 0.24) (Fig. 2d). No statistical difference in mean relatedness was found between year cohorts. The greatest difference was seen on Escape Island where r decreased from 0.24 (2014) to – 0.05 (2018), however, the sample size of the 2018 cohort was small (n = 5) (Fig. 2d).

**Table 2:** Genetic diversity in dibblers on Boullanger, Whitlock, and Escape Islands.

Population	N	NA (± se)	N_AR_ (± se)	H_O_ (± se)	H_E_ (± se)	F_IS_ (± se)	Ne (95% CI*)	Bottleneck
Boullanger Island								
2013	23	2.00 (0.21)	1.87 (0.05)	0.31 (0.06)	0.32 (0.06)	0.04 (0.02)	6.6 (1.7–50.9)	Y
2014	21	1.86 (0.21)	1.74 (0.04)	0.31 (0.08)	0.29 (0.06)	– 0.05 (0.03)	7.6 (1.9–65.3)	Y
2015	18	1.86 (0.21)	1.72 (0.04)	0.26 (0.07)	0.26 (0.06)	0.06 (0.02)	2.7 (1.6–7.9)	N
2016	25	1.86 (0.21)	1.77 (0.05)	0.30 (0.29)	0.29 (0.07)	− 0.02 (0.02)	3.1 (1.4–13.7)	Y
2017	32	1.86 (0.21)	1.73 (0.04)	0.29 (0.06)	0.28 (0.06)	− 0.07 (0.02)	3.9 (2.4–10.9)	Y
Whitlock Island								
2013	20	1.14 (0.10)	1.13 (0.02)	0.05 (0.03)	0.05 (0.03)	0.00 (0.11)	∞ (0.0–∞)	N
2014	18	1.21 (0.11)	1.14 (0.02)	0.04 (0.02)	0.04 (0.03)	0.01 (0.04)	∞ (0.0–∞)	N
2015	22	1.21 (0.11)	1.15 (0.02)	0.05 (0.03)	0.05 (0.03)	0.11 (0.15)	22.6 (0.0–∞)	N
2016	24	1.14 (0.10)	1.13 (0.02)	0.04 (0.03)	0.04 (0.03)	− 0.03 (0.10)	0.6 (0.1–2.0)	N
2017	25	1.14 (0.10)	1.13 (0.02)	0.05 (0.03)	0.05 (0.03)	− 0.06 (0.06)	∞ (1.7–∞)	N
2018	9	1.07 (0.07)	1.07 (0.02)	0.02 (0.02)	0.02 (0.02)	− 0.14 (0.00)	∞ (∞–∞)	N
Escape Island								
2014	20	2.07 (0.20)	1.99 (0.04)	0.42 (0.06)	0.38 (0.05)	− 0.08 (0.02)	17.6 (3.4–∞)	Y
2018	5	2.00 (0.18)	2.00 (0.05)	0.37 (0.06)	0.36 (0.05)	0.04 (0.03)	17.8 (1.5–∞)	N

N, number of individuals with genotypes; NA, mean number of alleles per locus; N_AR_, allelic richness; H_O_, observed heterozygosity; H_E_, expected heterozygosity; F_IS_, inbreeding coefficient; Ne, effective population size. Standard errors for means or 95% confidence limits are presented in brackets. 95% CI values were estimated by jackknife re-sampling.

Pairwise F_ST_ and pairwise Jost’s D values indicated that all populations were significantly differentiated from each other (*P* < 0.01) (Table 3). The Escape Island population had a smaller genetic distance to Boullanger Island, relative to its genetic distance to Whitlock Island (Table 3). Clustering and ΔK analysis showed that the island populations form two distinct genetic groups (*K* = 2), and models that included admixture showed better convergence and greater absolute probabilities (mean posterior probability (± s.d.) of − 2435.8 (± 0.6) with admixture vs. − 2540.5 (± 1.9) without admixture). Despite an even number of founders used in the captive breeding program plus an additional three Whitlock island males^46^, the admixture seemed to bias toward the Boullanger source population (Fig. 1b). The mean (± s.d.) per individual contribution from Boullanger Island in the 2014 cohort was 69.3% (± 0.2), and 14 out of 20 individuals had > 50% Boullanger contribution (i.e. 90% credible intervals above a Q score of 0.5). This was reduced to 54.7% (± 0.8) in the 2018 cohort, and 1 out of 5 individuals had > 50% Boullanger contribution, although the sample size for this latter cohort was low (n = 5) (Fig. 1b).

**Table 3:** Pairwise distance values for dibblers from Boullanger, Whitlock, and Escape Islands.

Population	Year	Boullanger Island					Whitlock Island						Escape Island	
		2013	2014	2015	2016	2017	2013	2014	2015	2016	2017	2018	2014	2018
Boullanger Island	2013		0.02	0.02	0.00	**0.04**	**0.58**	**0.58**	**0.59**	**0.61**	**0.61**	**0.55**	**0.23**	**0.27**
	2014	0.01		0.02	0.00	0.02	**0.58**	**0.58**	**0.59**	**0.61**	**0.61**	**0.56**	**0.28**	**0.30**
	2015	0.01	0.01		− 0.01	0.00	**0.60**	**0.61**	**0.61**	**0.64**	**0.64**	**0.58**	**0.27**	**0.31**
	2016	0.00	0.00	0.00		0.02	**0.57**	**0.58**	**0.58**	**0.60**	**0.60**	**0.55**	**0.27**	**0.30**
	2017	**0.02**	0.01	0.00	0.01		**0.53**	**0.54**	**0.54**	**0.56**	**0.56**	**0.52**	**0.27**	**0.29**
Whitlock Island	2013	**0.33**	**0.29**	**0.28**	**0.30**	**0.26**		− 0.01	− 0.01	− 0.01	0.03	0.09	**0.44**	**0.47**
	2014	**0.34**	**0.30**	**0.29**	**0.31**	**0.28**	0.00		− 0.01	− 0.02	0.03	0.04	**0.44**	**0.48**
	2015	**0.34**	**0.29**	**0.28**	**0.31**	**0.27**	0.00	0.00		− 0.02	− 0.01	0.03	**0.45**	**0.48**
	2016	**0.34**	**0.30**	**0.29**	**0.32**	**0.28**	0.00	0.00	0.00		0.00	0.02	**0.47**	**0.52**
	2017	**0.34**	**0.30**	**0.29**	**0.32**	**0.28**	0.00	0.00	0.00	0.00		0.02	**0.47**	**0.52**
	2018	**0.36**	**0.32**	**0.31**	**0.34**	**0.30**	0.00	0.00	0.00	0.00	0.00		**0.40**	**0.42**
Escape Island	2014	**0.17**	**0.20**	**0.19**	**0.19**	**0.18**	**0.22**	**0.22**	**0.22**	**0.23**	**0.23**	**0.24**		− 0.02
	2018	**0.19**	**0.20**	**0.20**	**0.20**	**0.18**	**0.12**	**0.12**	**0.13**	**0.13**	**0.13**	**0.14**	− 0.02	

Pairwise F_ST_ values are above diagonal and pairwise Jost’s D below the diagonal. Values significantly greater than zero (*P* < 0.01) after correction for multiple comparisons are shown in bold.

Boullanger and Escape Island populations showed significant deviation from the mutation-drift equilibrium (Wilcoxon’s one-tailed test; *P* < 0.05), suggesting that a bottleneck event has occurred recently within these populations (Table 2). No significant bottleneck event was detected in the Whitlock Island population (Table 2), although it is not possible to generate a reliable probability based on only one polymorphic locus with a one-tailed Wilcoxon sign rank test or any tests run in BOTTLENECK^62^. Estimates of effective population size (Ne) were low for Boullanger and Escape Islands but could not be determined for most Whitlock Island populations (Table 2). The upper confidence intervals of infinity in Whitlock and Escape Island populations (Table 2) were likely due to low power resulting from low sample size (Escape Island 2018), small number of polymorphic markers (Whitlock Island all years) or a combination of both (Whitlock Island 2018)^70^.

### Population viability analysis

The deterministic growth rate (lambda) of the Boullanger Island population was high and comparable to that of the Escape Island population (1.68 and 1.62 respectively), while the Whitlock Island population had the lowest lambda at 1.49. The cohort generation times (Tc) were estimated to be 1.53 (Boullanger Island), 1.56 (Whitlock Island) and 1.54 (Escape Island), with a mean Tc across populations of 1.54 years.

**Figure 3:**
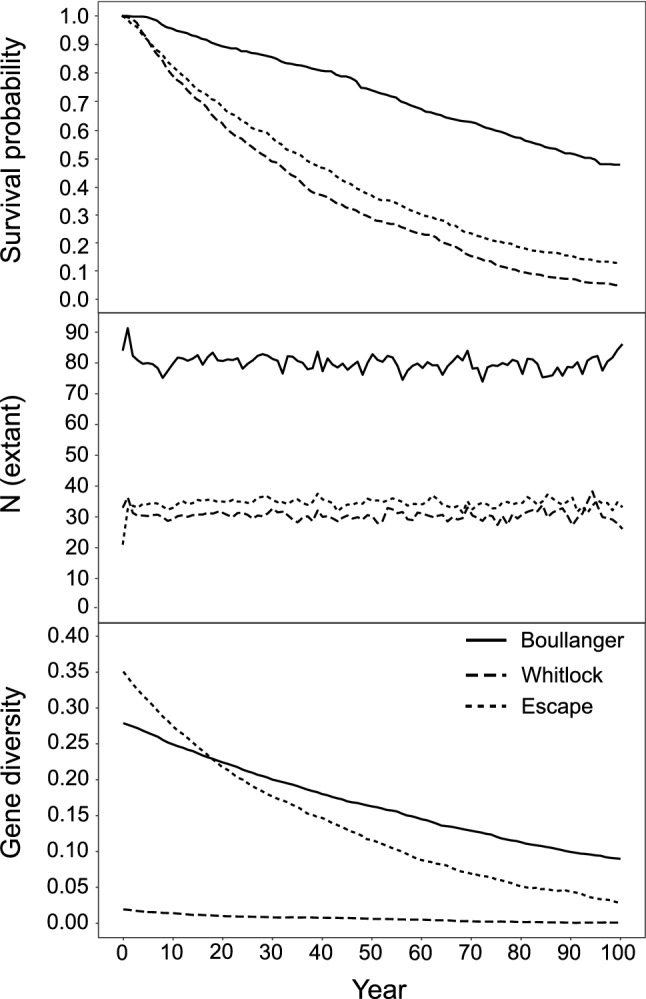
One hundred-year projections of survival probability, population size and gene diversity for the dibbler populations on Boullanger (solid line), Whitlock (large dashes), and Escape (small dashes) Islands.

The baseline model predicted that for those populations that survive 100 years (or around 65 generations), Boullanger Island’s population size remained relatively stable, Whitlock Island’s population decreased (from 33 to 26) and Escape Island’s population increased (from 21 to 33) (Fig. 3). However, extinction rates for all three islands were high and increased over time. Population simulations predicted the average time to extinction for Boullanger, Whitlock and Escape Islands were 50, 34 and 35 years, respectively. This equates to, in order, 32, 22 and 23 generations. The probability of survival after 100 years was 48% for the Boullanger Island population, 13% for the Escape Island population and 6% for the Whitlock Island population. Declines in the Boullanger Island survival probabilities over time were more gradual compared to Whitlock and Escape Islands (Fig. 3). Genetic diversity was also projected to decline over time, again more gradually in Boullanger Island, and after 100 years all populations were estimated to have very low gene diversity (< 0.1; Fig. 3), high observed homozygosity (> 0.9), and reduced numbers of alleles per locus (< 2).

Linear regression on sensitivity testing showed that the frequency of droughts has the greatest impact on the survival of new dibbler populations (R^2^ = 0.97, *P* < 0.001) while carrying capacity also has a strong effect (R^2^ = 0.86, *P* < 0.001). Founder size did not have a strong impact on survival. Sensitivity analyses are summarised in Supplementary Fig. S2.

#### Models of recent population dynamics

Estimates of population size, gene diversity and number of alleles in 2018 for all three island populations from a six-year (~ four generations) PVA model populated with 2012 empirical data were in good agreement with observed values (Tables 2 and 4). Modelling estimates of H_E_ and NA for Boullanger and Escape Islands (Table 4) fell within the error margins for empirical estimates (Table 2). Modelling estimates for both H_E_ and NA in the 2018 Whitlock Island cohort were slightly greater than the upper error margin from empirical data, however, the 2018 Whitlock Island population had a small sample size (n = 9) and only one polymorphic locus, increasing error in the empirical estimates. Nonetheless, both modelling data and empirical data show the 2018 trend of highest genetic diversity in Escape Island, closely followed by Boullanger Island with substantially lower genetic diversity in Whitlock Island. The six-year probability of survival was 98% in Boullanger Island, 91% in Escape Island and 90% in Whitlock Island.

**Table 4:** Population parameters for island dibblers in 2018 estimated by population viability analysis.

Island	Population size (± sd)	NA (± sd)	H_E_ (± sd)	Observed homozygosity (± sd)
Boullanger	80.2 (32.6)	1.87 (0.06)	0.30 (0.02)	0.70 (0.03)
Whitlock	31.4 (16.1)	1.17 (0.05)	0.05 (0.02)	0.95 (0.02)
Escape	35.1 (16.3)	1.90 (0.10)	0.32 (0.04)	0.66 (0.04)

NA: mean number of alleles, HE: expected heterozygosity.

#### Models of harvesting and translocation scenarios

The best scenario for establishing a captive breeding program of ten breeding pairs from the parental populations was to harvest 14 dibblers (7 males, 7 females) from Boullanger Island or Escape Island, and six dibblers (3 males, 3 females) from Whitlock Island (scenario 8, Supplementary Table S3). This scenario maintained a high probability of survival in both source populations in the years immediately following harvest and produced the highest genetic diversity for the captive bred population. While harvesting more than 14 individuals from Escape Island slightly increased genetic diversity in the captive population, survival of the Escape Island population drops sharply when more than 14 individuals are harvested.

Based on these translocation scenarios, releasing 80 individuals from the captive breeding program on Dirk Hartog Island over two years (40 per year) should be the minimum threshold due to the survival probability of above 90% and slightly higher predicted levels of gene diversity (for both source populations combinations, Table 5a). However, when Boullanger and Whitlock Island populations are used as sources, it produces slightly higher survival probability and gene diversity. The initial gene diversity of the Dirk Hartog Island population is predicted to be higher than both source populations. If Escape and Whitlock Island populations are used as sources, the initial gene diversity of the Dirk Hartog Island population is predicted to be only higher than Whitlock Island, and slightly lower than Escape Island. However, after 100 years, with either source island combination gene diversity of dibblers on Dirk Hartog Island is predicted to be substantially higher (0.26 or 0.27, Table 5a) than all island populations (0.00 to 0.09) due to a larger area and carrying capacity of Dirk Hartog Island (Fig. 3).

**Table 5:** Impact of founder size on reintroduced population of dibblers from captive breeding after 100 years, using (a) optimal captive breeding harvest scenarios from Supplementary Table S3 (Scenario 8) and (b) the current harvest involving nine individuals from Whitlock Island and five individuals from Escape Island.

(a)						
Source populations	N_founder_	60	80	100	120	140
Boullanger and Whitlock Islands	N_ext_	7577	7665	7834	7692	7710
	P(survival)	0.89	0.91	0.94	0.93	0.94
	Gene Diversity	0.26	0.27	0.27	0.27	0.27
	Observed homozygosity	0.74	0.73	0.73	0.73	0.73
Escape and Whitlock Islands	N_ext_	7778	7729	8005	7936	7547
	P(survival)	0.89	0.89	0.87	0.89	0.90
	Gene Diversity	0.25	0.26	0.26	0.26	0.26
	Observed homozygosity	0.75	0.74	0.74	0.74	0.74

(b)						
N_founder_	40	60	80	100		
N_ext_	7229	7768	7457	7787		
P(survival)	0.77	0.79	0.81	0.81		
Gene diversity	0.16	0.16	0.16	0.17		
Observed homozygosity	0.84	0.84	0.84	0.83		

N_founder_ is the number of translocated individuals; N_ext_ is the number of individuals expected in a surviving population after 100 years; gene diversity is equivalent to expected heterozygosity (H_E_).

#### Modelling the current captive-breeding and release program

A model that simulated the recent harvest of island populations for captive breeding (nine individuals from Whitlock Island and five individuals from Escape Island) showed a minimum of 80 dibblers should be released from the captive breeding program over two years (40 per year) on Dirk Hartog Island (Table 5b), as survival probability exceeds 80%. However, this projected probability of survival is lower than for the recommended scenarios across all founder sizes (Table 5a). With 80 founders, at year one post-translocation gene diversity of the Dirk Hartog Island population is projected to be initially higher (0.17) than the gene diversity of the Whitlock Island source population (0.02) and not higher than the Boullanger (0.27) or Escape Island (0.32). However, gene diversity is predicted be higher than all source populations after 100 years, but still 38.4% lower than the optimal model (Table 5a).

## Discussion

Since European settlement in the early 1800s Australia has lost much of its faunal diversity, and a further 106 mammalian species are at risk of extinction^71^. Dibblers are a prime example of these vulnerable species, as we predict that Jurien Bay island populations will decline over the next century. The use of PVA has provided important insights into the genetic and ecological management of the Jurien Bay island populations, as well as a framework for planning translocations to Dirk Hartog Island, which if successful, will provide a critical insurance population where the relictual genetic variation can be maintained.

### Current genetic variation within island dibbler populations

Low genetic variation is common in island populations of many mammal species^72,73^, in line with the results of this study. Genetic drift is expected to have a large impact on the genetic diversity of island dibblers, as smaller populations are more prone to drift than larger ones^73,74^. Drift decreases heterozygosity at the rate of $\frac{1}{2Ne}$^74^, and if population sizes remain small for multiple generations, large losses of heterozygosity are more likely^6^. While we were not able to show a significant reduction in the various genetic diversity measurements we used across our year cohorts for each island, which spanned a 4 or 5-year period, in almost every case the most recent estimate was less than the initial estimate (Table 2). It is possible that insufficient time has passed for temporal reductions in genetic diversity. However, when heterozygosity estimates from this study are compared to a previous study based on the same genetic markers^39^, there is a greater apparent reduction in all three Jurien Bay island populations—Boullanger Island (~ 30%) and Whitlock Island (~ 72%) from 2006 to 2017, and Escape Island (~ 12%) from 2002 to 2018. This suggests a gradual loss of genetic diversity, which is not unexpected as islands are isolated with no gene flow, however this finding requires formal testing.

Effective population size (Ne) estimates for island dibblers were small or undefined with evidence that two of the three island populations have undergone recent bottleneck events. However, facultative male die-off on Boullanger Island may cause an overestimation of recent bottlenecking events and underestimates of Ne. Therefore, the small estimated Ne for Boullanger Island across cohorts may be misleading. Infinite estimates of Ne generally suggest very large population sizes^70,75^, however, this cannot be the case for Whitlock Island given the carrying capacity is 42. As more than five polymorphic loci are required to accurately predict Ne values of 100 using the linkage disequilibrium method^70^, infinite estimates for Whitlock Island likely reflect the low power from the low number of polymorphic loci, for example there were only two polymorphic loci in the 2017 cohort. Overall, although there is uncertainty around estimates, the Ne values for all island populations are likely to be much smaller than Ne values recommended to avoid inbreeding depression (Ne ≥ 100)^76^. This suggests that these island populations are more prone to the effects of genetic drift and loss of heterozygosity, and are susceptible to inbreeding depression and the loss of adaptive potential^14^.

Escape Island has the highest genetic diversity due to the population being admixed from Boullanger and Whitlock Islands^36^. Like other studies^77–79^, this endorses the use of multiple source populations to found translocated populations, as translocated populations often have higher genetic variation than their respective source populations. Furthermore, the higher individual heterozygosity observed in Escape Island dibblers relative to dibblers on other islands indicate this mixed population may have higher fitness^80–82^.

Interestingly, we provide tentative support that the 2014 population on Escape Island may have greater genomic representation from Boullanger Island than Whitlock Island. Genetic distances (F_ST_ and Jost’s D) were also greater between Whitlock and Escape Islands than between Boullanger and Escape Islands in 2014, but interpretation of this trend is confounded by the level of fixation in Whitlock. If this representation bias in the Escape Island population towards Boullanger Island is real, it has occurred despite equal numbers from both parental populations being used to found the original captive bred source population^36^, and requires further investigation. Males from Boullanger Island are larger than males from Whitlock Island^32,39^, and heavier and younger males were observed to have higher mating success in captivity^46,83^. As biased mating leads to a decrease in heterozygosity, an increase in inbreeding and a reduction in Ne^84^, confirmation or refutation of this trend in the wild would be useful to help future translocations maximise retention of genetic diversity.

Whitlock Island dibblers were found on average to be highly related to each other (*r* > 0.8), implying high levels of inbreeding and a deficit in heterozygotes. In contrast, negative inbreeding coefficients (F_IS_) in the 2017 and 2018 Whitlock Island populations reflected an unexpectedly high heterozygosity, which could be caused by a recent population bottleneck, inbreeding avoidance or biased sampling. Ambiguities in results for Whitlock Island are likely further confounded by the low power associated with few polymorphic loci available for this population (e.g. 3 loci in 2016 and 1 locus in 2018), likely due to fixation, in conjunction with the small sample size in 2018 (n = 9). Future studies with orders of magnitude more genome-wide genetic markers, e.g. single nucleotide polymorphisms (SNPs), will provide greater statistical power to better resolve whether inbreeding is occurring^85^, estimate effective population sizes^86^ and quantify inter-population genetic distances^87^.

### Long term viability of current and future populations of island dibblers

Small isolated populations are susceptible to reductions in survival and gene diversity, especially in the absence of immigration (i.e. gene flow)^73,88–90^. They are also particularly susceptible to stochastic environmental factors, compared to larger populations or those reared in benign environments such as in captive breeding facilities^91,92^. Here, we show that the survival probabilities and genetic diversities of dibbler populations on the Jurien Bay Islands are predicted to decline over time, and the smaller populations (Whitlock and Escape Islands) will likely expire within 50 years, or around 33 generations. These observations are consistent with observed downward population trajectories (J.A. Friend, personal observation) and the apparent gradual reductions in genetic diversity we see when this study is compared to Thavornkanlapachai^39^.

Island dibbler populations appear to be particularly sensitive to carrying capacity and the frequency of drought. Although the deterministic growth rates are relatively high, so too are the estimated rates of juvenile and adult mortality. High juvenile mortality is typical of mammals that mature early and produce large numbers of offspring after a short gestation^93^. If stochastic events such as drought occur more frequently, temporarily reducing reproduction and increasing mortality, and population recovery is constrained by limited carrying capacities, population sizes may fall below extinction thresholds. Concerningly, drought events will likely become increasingly severe as periods of low rainfall become more frequent in the future^94,95^. To compound matters, due to its extensive sand-based habitat, Boullanger Island is projected to erode as sea levels rise and further reduce its carrying capacity (J.A. Friend, personal observation). In contrast, the carrying capacities for Whitlock and Escape Islands are not expected to change over the next century, but the small size of these islands limits population growth, and so could substantially constrain long-term viability^96^.

With an estimated carrying capacity for dibblers of 10,000 on Dirk Hartog Island, the inclusion of dibblers as part of the ‘Return to 1616’ ecological restoration initiative will be critical for their long-term viability. As genetic variation in island dibblers is low and declining, genetic management will be required to prevent further loss. Our PVA modelling indicates admixing island populations in a captive breeding program will maximise the genetic diversity and subsequent adaptive potential of the introduced population on Dirk Hartog Island. If the population establishes successfully, the ideal founder population (Scenario 8) will be able to capture higher genetic diversity relative to single source populations, and the predicted growth of the Dirk Hartog Island population to a census size above 6000 will facilitate the retention of this diversity.

### Recommendations for dibbler management

The benefits of using PVA to optimise translocation designs have been demonstrated in many other threatened vertebrates, including banded hare-wallabies^44^, woylies^43^, golden bandicoots^97^, pygmy rabbits^98^ and frogs^15^. The good agreement between historical empirical data and our simulations gives confidence that our models can predict demographic and genetic changes relatively accurately. We have therefore provided a management tool that can help predict outcomes of conservation actions, even in the absence of further empirical data. Furthermore, our sensitivity analyses show that the viability of dibbler populations are strongly influenced by carrying capacity and the frequency of drought. Consequently, these ecological factors warrant inclusion in future conservation management of the island dibbler, and it is worthwhile to investigate ways in which to mitigate their impact. For example, the planting of preferred habitat vegetation may increase otherwise restricted local carrying capacity, and the provision of alternate sources of water could help traverse challenging drought periods^99,100^, which for dibblers would mean supplementing insect prey. To this end, introductions to Dirk Hartog Island will facilitate a much greater carrying capacity than exists on any of the Jurien Bay islands.

The current captive breeding program implemented at Perth Zoo should result in higher genetic diversity in the new Dirk Hartog Island population, relative to the founder populations, and reach survival probabilities of 80% if 80 individuals or more can be released over two years. Harvesting these numbers would not be feasible without a captive breeding program due to the very low current census sizes of the Jurien Bay island populations. However, the program would benefit from replacing three Whitlock Island dibblers with nine Escape Island dibblers. This would increase genetic diversity in the translocated population by 53% and reduce the threat of overharvesting on Whitlock Island. Due to downward population trajectories and the sensitivity of population viability on Whitlock Island to population crashes, we strongly advocate that harvesting is avoided during drought-driven periodic declines. Further, we recommend ongoing genetic monitoring of Jurien Bay island populations and the new Dirk Hartog Island population to detect potential reductions in genetic diversity and increases in inbreeding, as well as monitoring for signs of inbreeding depression.

To improve population viability on Boullanger, Whitlock and Escape Islands, as well as in future translocated populations, it is worth considering demographic and genetic augmentation via supplementation from dibbler populations that occur on the Western Australian mainland (e.g. Fitzgerald River National Park or Peniup Nature Reserve). Mainland population sizes are larger and more genetically diverse than those on the Jurien Bay islands^29,32,39^, and should therefore be more robust to harvesting. Using them to supplement island dibblers could lead to a genetic rescue effect, which would increase genetic diversity, fitness and evolutionary potential, ultimately reducing the risk of extinction^21,101–106^. However, several studies have found that crossing genetically distinct populations or subspecies can reduce the overall fecundity and viability of species by both pre- and post-zygotic reproductive barriers^107–109^. For example, outbreeding depression may occur where hybrid offspring are maladapted to local environments and consequently suffer reductions in fitness^10,26–28^. The Jurien Bay Islands have been separated from the mainland for over 6500 years^110^, and presumably a greater time still has passed since gene flow occurred between the Jurien Bay dibbler populations and those 600 km away on the south coast of Western Australia. What’s more, the two regions have different climates and habitats. Consequently, mainland dibblers have begun to diverge from island dibblers—for example, mainland dibblers are significantly larger in body size and weight than island dibblers^32,33^ meaning non-random mating could potentially occur if mainland and island dibblers were to inter-breed^39,46^. In addition, island dibblers have a shorter breeding season and gestation period relative to mainland dibblers, as well as mate and produce offspring later in the year^29,31,47^. Hence, while there is an obvious rationale to mixing island and mainland dibblers to address low genetic diversity, we recommend the use of ex situ husbandry to determine the success of interbreeding between island and mainland animals before this becomes a recommended management option.

In summary, this study shows captive breeding and translocation are critical for continuation of the declining island dibblers. We have demonstrated how using both PVA models and genetic information can generate recommendations for ongoing and future conservation actions, e.g. conservation translocations, for threatened species such as the dibbler, and maximise their chances of success. The PVA model developed in this study, complemented with greater genetic resolution from using a substantially greater number of markers (e.g. SNPs), should be used to evaluate future viability of the translocated population on Dirk Hartog Island, to better inform ongoing management of the species. After quantifying the probability and impact of outbreeding depression, expanding the modelling developed here to encompass all extant dibbler populations (i.e. island and mainland) will be useful for predicting the long-term consequences of admixture between the more distantly related source populations. With many threatened Australian species experiencing increased fragmentation and subsequently elevated extinction risk, devising strategies that maximise genetic diversity, such as admixture, is increasingly being considered for long-term management.

## Supplementary Information

Supplementary Information.

## Data Availability

Genotypes for all 2013 or later samples are stored in a database at the Department of Biodiversity, Conservation and Attractions and are available upon request.
