# Supplementary material for: Using PVA and captive breeding to balance trade-offs in the rescue of the island dibbler onto a new island ark
Source: Sci Rep. 2022 Jul 13;12:11913. doi: 10.1038/s41598-022-14150-9 (PMC9279492; doi:10.1038/s41598-022-14150-9)
Supplement: Supplementary file 1 — Supplementary Information. [file 41598_2022_14150_MOESM1_ESM.pdf]

Using PVA and captive breeding to balance trade-offs in the rescue of the island dibbler onto a new island ark

Zahra Aisya[a], Daniel J. White*[a], Rujiporn Thavornkanlapachai[b], J. Anthony Friend[c], Kate Rick[a], Nicola J. Mitchell[a]

[a] School of Biological Sciences, The University of Western Australia, Crawley, Western Australia 6009, Australia

[b] Department of Biodiversity, Conservation and Attractions, Locked Bag 104, Bentley Delivery Centre, Western Australia 6152, Australia

[c] Department of Biodiversity, Conservation and Attractions, 120 Albany Highway, Albany, WA 6330, Australia

*Corresponding author: Daniel White

School of Biological Sciences, The University of Western Australia

35 Stirling Highway, Perth WA, 6009, Australia

Phone: +61 451 675 069

Email: daniel.white@uwa.edu.au

Supplementary Information

Supplementary Table S1 The 14 microsatellites used to characterise genetic diversity within island populations of dibblers.

Locus	Origin (species)	Repeat	Size (bp)	Multi-plex	Anneal Temp (^oC)	Primer sequence (5'-3')	Reference
Aa4A	*Antechinus agilis*	NA	165-167	1	46	F: TTTGATCCTCAGAGACTTGAT<br>R: CCAAATCTACGTAAAATATCC	[1]
DG1A1	*Dasyurus geoffroii*	$(AC)_{20}$	197-227	3	54	F: ATTTGCTTCTTGCTCCCTACAGC<br>R: TTTCACTCCTTCTGAGTTTATCACC	[2]
3.1.2	*Dasyurus spp.*	$(CA)_{18}$	143-169	2	52	F: AGGAAACTTCACAAGTGTCGA<br>R: ATTAATGACTCATCTGTTGTTGG	[3]
3.3.1	*Dasyurus spp.*	$(CA)_{20}$	91-145	3	54	F: CAGCCCTTGAGTCTTGAGATT<br>R: CATACCACCCCAGGAGTTTC	[3]
3.3.2	*Dasyurus spp.*	$(CA)_{21}$	108-148	1	46	F: AATAGCAGAGACTCGATCC<br>R: AGCCTTTATTACCTGGGAAG	[3]
4.4.2	*Dasyurus spp.*	$(CA)_{19}$	70-110	1	56	F: GAAATCCAAGCTCATTTTAG<br>R: AATCAACTCTGGAATGCATC	[3]
4.4.10	*Dasyurus spp.*	$(CA)_{29}$	179-217	3	54	F: AATGCTAGATTTCACTCCC<br>R: CCTCACATTTCTGGAACTG	[3]
Pa1B10	*Parantechinus apicalis*	$(GAAA)_{46}$	277	4	58	F: AAGGAGGGATGGAGGAGGAA<br>R: CAGTGTTCGAATGACATTGGCTAC	[4]

Pa2A12	*P. apicalis*	$(GT)_{21}$	131	2	56	F: ATCCTGGAGAAGAGAAGACCTGC<br>R: GTGGCTTATTCCATGCTTGTAGG	4
Pa2B10	*P. apicalis*	$(GT)_{23}$	213	4	58	F: GAGAAAAAATATGCACAAGCACC<br>R: AAGGAGAAAAAGTTAATACCATCCC	4
Pa2D4	*P. apicalis*	NA	193-197	2	56	F: CAATCTGTCAATAACCTTCCCCC<br>R: TGGAGGACCTCCAGAAAGTTAGC	4
Pa4B3	*P. apicalis*	$(GT)_{15}$	121	4	58	F: GAAGGACAACATTCCCGATTGT<br>R: CCTACCCTAATTGCAAATCCTTTC	4
Pa7A1	*P. apicalis*	$(GAA)_{85}$	300	2	56	F: CTCCACCTCTCTAGACATGACCC<br>R: TTTACTTGCTTTGTACTAGAGGCC	4
Sh6e	*Sarcophilus laniarius*	$(CA)_6$<br>$(A)_2(CA)_{18}$	203-217	3	54	F: GATTCTAGAAGGGATAGCAAGC<br>R: GACACTCCATAGAAATGCACTG	5

Supplementary Table S2 Demographic and life history traits of dibblers from Boullanger (BO), Whitlock (WH), and Escape (ES) Islands, as well as the proposed captive breeding program (CB) and Dirk Hartog (DH) Island populations, used in population viability analysis within the software VORTEX [6]. NA denotes not applicable. A description for each of the parameters are detailed below the table.

**Parameter**	***Parantechinus apicalis***
Scenario Settings	
Number of iterations	500
Number of years (timesteps)	100
Duration of each year in days	365
Extinction definition	Only one sex remains
Number of populations	BO = 1, WH = 1, ES = 1, CB = 1, DH = 1
Species Description	
Inbreeding depression	
- Lethal equivalents	NA
- Percent due to recessive lethal alleles	NA
EV correlation between reproduction and survival	0.5 (default)
EV correlation among populations	0.5 (default)
Dispersal	NA (closed populations)
Reproductive Systems	
Reproductive system	Polygynous
Age of first offspring	1
Maximum age of reproduction	3
Maximum lifespan	3
Maximum number of broods per year	1
Maximum number of progeny per brood	8
Sex ratio at birth – in % males	49.7
Reproductive Rates	
% adult females breeding	90
SD in % breeding due to EV	10

Distribution of number of offspring per female per brood	
- Mean	BO = 7.4, WH = 6.2,<br>ES & DH = 7.0, CB = 7.12
- Standard Dev	BO = 0.1, WH = 0.23,<br>ES & DH = 1.1, CB = 1.17
Mortality rates	
Mortality of females as %	
- Mortality from age 0-1 (± SD)	BO, WH, ES, DH = 59 ± 10<br>CB = 29.4 ± 10
- Annual mortality after age 1 (± SD)	35 ± 10
Mortality of males as %	
- Mortality from age 0-1 (± SD)	BO, WH, ES, DH = 59 ± 10<br>CB = 29.4 ± 10
- Annual mortality after age 1 (± SD)	BO = (((Y%8)<1)*65)+35 ± 10<br>WH, ES, CB, DH = 35 ± 10
Catastrophes	
Number of types of catastrophes	1
Frequency and extent of occurrence	
- Frequency %	BO, WH, ES, DH = 12.5<br>CB = 0
Severity (proportion of normal values)	
- Reproduction	BO, WH, ES, DH = 0.3<br>CB = 1
- Survival	BO, WH, ES, DH = 0.3<br>CB = 1
Mate Monopolisation	
% Males in breeding pool	100
Initial Population Size	
Initial Population Size	BO = 84, WH = 33,<br>ES = 21, CB & DH = 0
Distribution	Use stable age distribution

Carrying Capacity	
Carrying Capacity (K)	BO = 100, WH = 42, ES = 45, CB = 80, DH = 10000
SD in K due to EV	BO = 13, WH = 12, ES = 12, CB = 0, DH = 1000
Genetics	
Number of neutral loci to be modelled	15
Loci included in summary statistics	Additional loci only

Scenario Settings: Though dibblers reach sexual maturity by the age of 10 months, they are seasonal breeders and will only breed once every year. This is why the duration of each year in days is set to 365 (one calendar year).

Species Description: It is uncertain how inbreeding affects dibblers, or whether inbreeding does affect populations. It was decided that with such small population numbers, if inbreeding were to have any deleterious effects, they would have been purged from populations. Therefore, inbreeding was not selected in the model, making the lethal equivalent and the percentage due to lethal alleles not applicable.

Reproductive Systems: As dibblers become sexually mature by the age of 10 months [7], the age of first offspring is set to 1 year. Values for sex ratio at birth in Boullanger and Whitlock Islands were provided by Tony Friend (pers. comm.), the value stated in this table is the mean of these two values.

Reproductive Rates: The percentage of females breeding was obtained from Moro [8]. As there were no available data on the environmental variation of female breeding, we have assumed a value of 10% to be the standard deviation. The distribution of number of offspring per brood for Boullanger and Whitlock Islands were obtained from Mills et al. [9]; for Escape Island from Moro [8]; for captive breeding from Lambert and Mills [10]. As Dirk Hartog Island will be a new translocated population, current data is unknown. We have assumed that Dirk Hartog Island will be similar to Escape Island, an established translocated population that was formed from captive breeding Boullanger and Whitlock Island dibblers.

Mortality Rates: The value for mortality from age 0-1 years was estimated from juvenile mortality in captive-bred populations [10]. The value presented for island populations was the captive-bred mortality multiplied by two. The estimated annual mortality rate of dibblers after age 1 was obtained from Moro

[8]. Facultative male die-off only occurs on Boullanger Island [7,11,12], and is modelled using the formula “=(((Y%8)<1)*65)+35”, which states that every 8th year, mortality of males will equal 100. As the frequency of facultative male die-off has not been quantified, we have assumed that the phenomenon occurs every eight years, based upon trends of low rainfall periods in the Jurien Bay region [13]. Because Dirk Hartog Island will be a new translocated population, current data is unknown. We have assumed that Dirk Hartog Island will be similar to the translocated population on Escape Island, in that there will be no facultative male die-off. As there is no available data regarding the environmental variation of mortality for dibblers, we have assumed the standard deviation of 10% for mortality in each age-group.

Catastrophes: Based on meteorological data of the Jurien Bay region [13], trends show low rainfall periods occurring on average every eight years. It is unknown how severe the impact of droughts are on dibblers, though from studies on related species agile antechinus[14] and brush-tailed phascogale [15], survival and reproduction post-drought was on average 30%.

Mate Monopolisation: As dibblers are polygynous, and with small island sizes, it is assumed that all sexually active males will breed in the breeding season.

Initial Population Size: Current estimates of population sizes is calculated from the most recent monitoring event in June 2018, where dibblers on Boullanger and Whitlock Islands were trapped (Tony Friend, pers. comm.). Boullanger: assumed recent facultative male die-off event has occurred; five females with pouch young were trapped, with population size estimates to be twice this number. Assuming ten females produce 74 pouch young (adult males died post-mating), initial population size would be 84. Whitlock: trapped 22 individuals, population estimated to be 1.5 times this number. As it is unknown whether females with pouch young were trapped, initial population was set to 33. Escape: has not been monitored in recent years but estimated to have around 20 individuals. Captive breeding population starts at zero in year 1, before being supplemented with ten mating pairs (20 individuals) in year 2. Dirk Hartog Island population starts at zero in year 1 and will be supplemented with a minimum of 60 individuals from a captive breeding population over years 3 and 4.

Carrying Capacity: The K for Whitlock Island was calculated from multiplying the largest known density of dibblers [7] with the size of the island. The K for Boullanger and Escape Islands were estimated based upon expert opinion (Tony Friend, pers. comm.). The SD in K for Boullanger and Whitlock Islands were calculated from the smallest known densities of dibblers [7] with the size of the islands. The SD in K for Escape Island mirrors Whitlock and is based upon expert opinion (Tony Friend, pers. comm.) on the similarity of Escape and Whitlock Islands. The K for captive breeding was provided by Cathy Lambert (pers. comm.) and does not have variation, as Perth Zoo can only hold eight mating pairs and 64 pouch young per annum. The K for Dirk Hartog Island is an estimate provided by experts (Saul Cowen, pers. comm.). The SD in K for Dirk Hartog Island is unknown, and is assumed to be 10% of the estimated K.

Genetics: Allele frequencies at 14 microsatellite loci were chosen to model genetic diversity. These were the same loci initially chosen by Thavornkanlapachai [16]. Because VORTEX models its own neutral loci, the number listed is 15 to account for the additional loci.

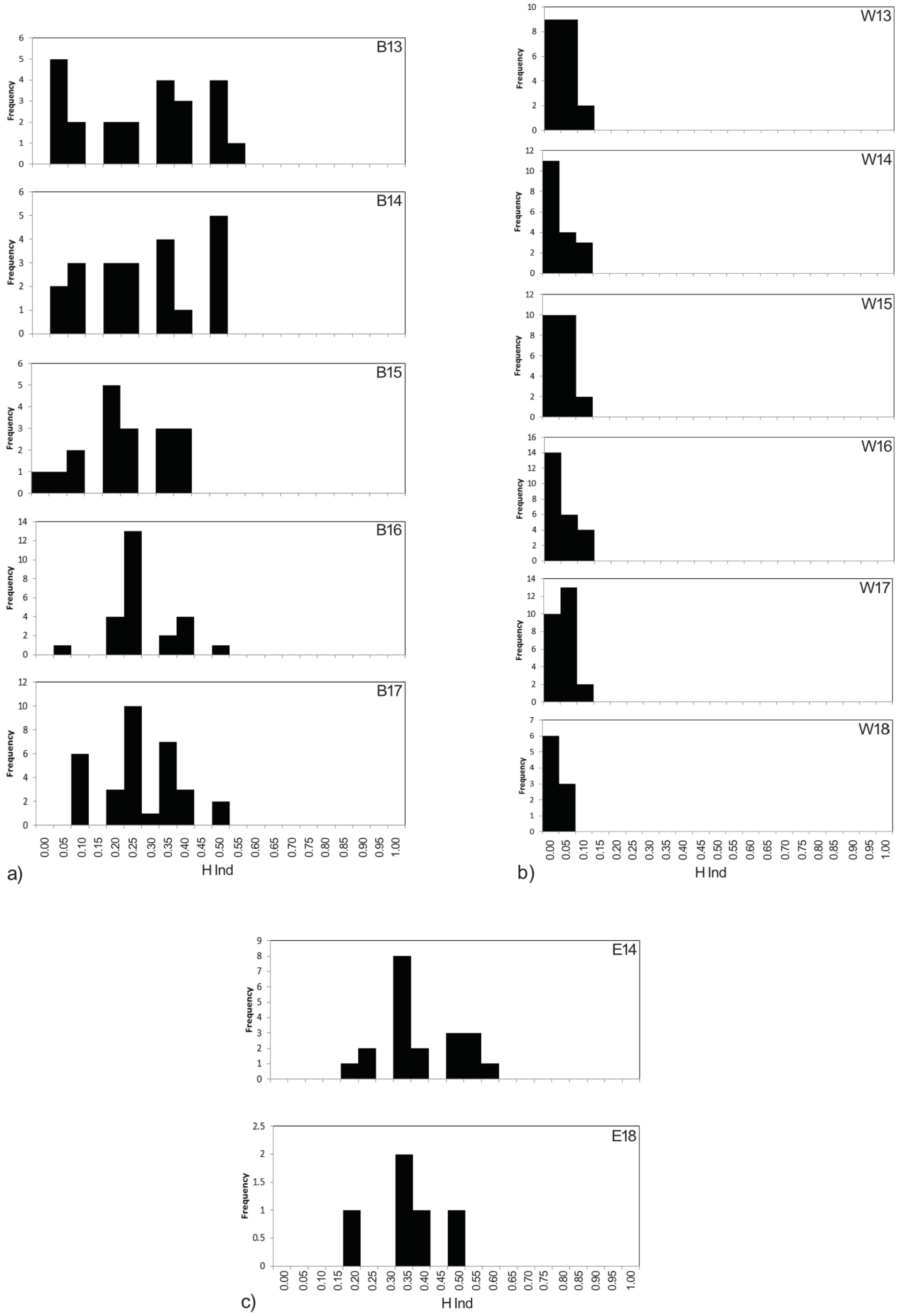

Supplementary Figure S1. Frequency distributions of individual heterozygosity across time cohorts of a) Boullanger Island, b) Whitlock Island and c) Escape Island Jurien Bay island dibblers. BXX: Boullanger Island and year; WXX: Whitlock Island and year; EXX: Escape Island and year.

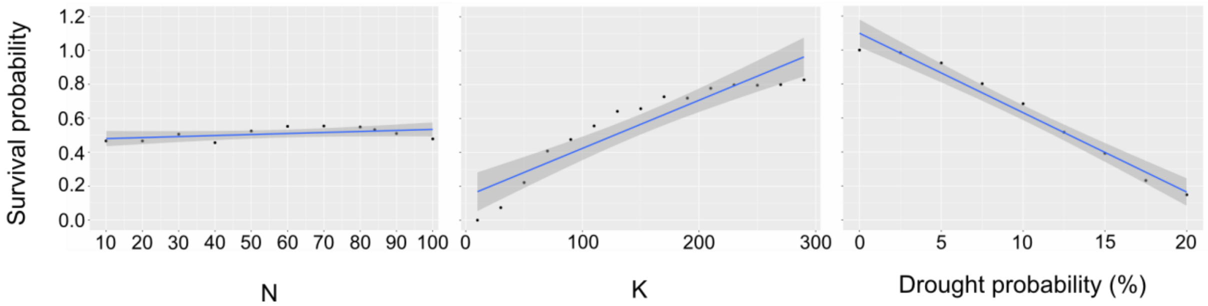

Supplementary Figure S2. Regression analysis on the impact of three demographic parameters on survival probability in island dibbler populations. $Survival\ probability = 0.0006 \times N + 0.4737, R^2 = 0.24, p = 0.126$; $survival\ probability = 0.0028 \times K + 0.1397, R^2 = 0.86, p < 0.001$; $survival\ probability = -0.0468 \times drought\ probability + 1.0989, R^2 = 0.97, p < 0.001$. Standard errors are shown (ribbons).

Supplementary Table S3 Impact of 11 harvesting scenarios for a captive breeding program on source populations. An equal sex ratio is assumed at harvest. Gene diversity is equivalent to expected heterozygosity. Numbers to the left of the forward slash represent translocations using Boullanger Island, whereas numbers to the right represent translocations using Escape Island.

Population	Scenario	SC1	SC2	SC3	SC4	SC5	SC6	SC7	SC8	SC9	SC10	SC11
Boullanger Island / Escape Island	# adults harvested	0	2	4	6	8	10	12	14	16	18	20
	P(Survival) one year post-harvest	1.00 / 0.98	1.00 / 0.95	1.00 / 0.93	1.00 / 0.89	1.00 / 0.88	1.00 / 0.88	0.99 / 0.87	0.98 / 0.88	0.97 / 0.80	0.96 / 0.83	0.95 / 0.75
	P(Survival) two years post-harvest	1.00 / 0.97	1.00 / 0.92	1.00 / 0.91	1.00 / 0.88	0.99 / 0.87	0.99 / 0.87	0.99 / 0.85	0.97 / 0.87	0.97 / 0.79	0.95 / 0.81	0.95 / 0.74
Whitlock Island	# adults harvested	20	18	16	14	12	10	8	6	4	2	0
	P(Survival) one year post-harvest	0.89 / 0.83	0.86 / 0.86	0.87 / 0.87	0.88 / 0.88	0.87 / 0.90	0.90 / 0.90	0.91 / 0.90	0.94 / 0.95	0.97 / 0.94	0.98 / 0.99	1.00 / 1.00
	P(Survival) two years post-harvest	0.88 / 0.83	0.85 / 0.85	0.87 / 0.86	0.87 / 0.87	0.87 / 0.89	0.89 / 0.88	0.90 / 0.89	0.91 / 0.94	0.96 / 0.92	0.95 / 0.97	0.99 / 0.98
Captive Breeding	Gene diversity one year post-founding	0.044 / 0.019	0.115 / 0.079	0.171 / 0.131	0.216 / 0.172	0.251 / 0.210	0.276 / 0.240	0.294 / 0.269	0.302 / 0.294	0.301 / 0.311	0.292 / 0.329	0.271 / 0.340

References

1 Banks, S. C. *et al.* The effects of habitat fragmentation due to forestry plantation establishment on the demography and genetic variation of a marsupial carnivore, Antechinus agilis. *Biological Conservation* **122**, 581-597, doi:10.1016/j.biocon.2004.09.013 (2005).

2 Spencer, P. B. S. *et al.* Cross-species amplification at microsatellite loci in Australian quolls including the description of five new markers from the Chuditch (Dasyurus geoffroii). *Molecular Ecology Notes* **7**, 1100-1103, doi:10.1111/j.1471-8286.2007.01791.x (2007).

3 Firestone, K. B. Isolation and characterization of microsatellites from carnivorous marsupials (Dasyuridae : Marsupialia). *Molecular Ecology* **8**, 1084-1086, doi:10.1046/j.1365-294X.1999.00655_6.x (1999).

4 Mills, H. R. & Spencer, P. B. S. Polymorphic microsatellites identified in an endangered dasyurid marsupial, the dibbler (Parantechinus apicalis). *Molecular Ecology Notes* **3**, 218-220, doi:10.1046/j.1471-8286.2003.00403.x (2003).

5 Jones, M. E., Paetkau, D., Geffen, E. & Moritz, C. Microsatellites for the Tasmanian devil (Sarcophilus laniarius). *Molecular Ecology Notes* **3**, 277-279, doi:10.1046/j.1471-8286.2003.00425.x (2003).

6 Lacy, R. C. & Pollak, J. P. VORTEX: A stochastic simulation of the extinction process. Version 10.0. (Brookfield, Illinois, USA, 2014).

7 Mills, H. R. & Bencini, R. New evidence for facultative male die-off in island populations of dibblers, *Parantechinus apicalis*. *Australian Journal of Zoology* **48**, 501-510, doi:10.1071/zo00025 (2000).

8 Moro, D. Translocation of captive-bred dibblers *Parantechinus apicalis* (Marsupialia : Dasyuridae) to Escape Island, Western Australia. *Biological Conservation* **111**, 305-315, doi:Pii s0006-3207(02)00296-310.1016/s0006-3207(02)00296-3 (2003).

9 Mills, H. R., Moro, D. & Spencer, P. B. S. Conservation significance of island versus mainland populations: a case study of dibblers (*Parantechinus apicalis*) in Western Australia. *Animal Conservation* **7**, 387-395, doi:10.1017/s1367943004001568 (2004).

10 Lambert, C. & Mills, H. Husbandry and breeding of the dibbler *Parantechinus apicalis* at Perth Zoo. *International Zoo Yearbook* **40**, 290-301 (2006).

11 Dickman, C. R. & Braithwaite, R. W. Postmating mortality of males in the Dasyurid marsupials, *Dasyurus* and *Parantechinus*. *Journal of Mammalogy* **73**, 143-147, doi:10.2307/1381875 (1992).

12 Wolfe, K. M., Robertson, H. & Bencini, R. The mating behaviour of the dibbler, *Parantechinus apicalis*, in captivity. *Australian Journal of Zoology* **48**, 541-550, doi:10.1071/zo00030 (2000).

13 Bureau of Meteorology, *Monthly rainfall Jurien Bay*, <http://www.bom.gov.au/jsp/ncc/cdio/weatherData/av?p_nccObsCode=139&p_display_type=dataFile&p_startYear=&p_c=&p_stn_num=009131> (2020).

14 Parrott, M. L., Ward, S. J., Temple-Smith, P. D. & Selwood, L. Effects of drought on weight, survival and breeding success of agile antechinus (*Antechinus agilis*), dusky antechinus (*A. swainsonii*) and bush rats (*Rattus fuscipes*). *Wildlife Research* **34**, 437-442, doi:10.1071/wr07071 (2007).

15 Rhind, S. G. & Bradley, J. S. The effect of drought on body size, growth and abundance of wild brush-tailed phascogales (*Phascogale tapoatafa*) in south-western Australia. *Wildlife Research* **29**, 235-245, doi:10.1071/wr01014 (2002).

16 Thavornkanlapachai, R. *Genetic consequences of genetic mixing in mammal translocations in Western Australia using case studies of burrowing bettongs and dibblers* Doctor of Philosophy thesis, University of Western Australia, (2016).
